# Clostridium perfringens Sepsis Complicated by Hepatic Abscess Following Intensive Chemotherapy in Mixed-Phenotype Acute Leukemia

**DOI:** 10.7759/cureus.77122

**Published:** 2025-01-08

**Authors:** Pedro Baptista, Marta Henriques, Mariana Miranda, Rui Bergantim, Fernanda Trigo

**Affiliations:** 1 Department of Clinical Hematology, Centro Hospitalar Universitário de São João, Porto, PRT; 2 Department of Clinical Hematology, Centro Hospitalar Tondela-Viseu, Viseu, PRT

**Keywords:** clostridium perfringens, febrile neutropenia, hepatic abscess, mixed-phenotype acute leukemia, severe sepsis

## Abstract

*Clostridium perfringens* (*C. perfringens*)* *is a rare cause of febrile neutropenia in patients with acute leukemia, and only a few case reports are published in the literature. The peculiar presentation of this clostridial sepsis, with the formation of gaseous liver abscess and toxin-associated intravascular hemolysis, can suggest the diagnosis in immunocompromised patients and prompt immediate treatment. Early identification of these clinical features may be essential to prevent the accelerated and aggressive course of this infection with a very high mortality rate. Here we report the case of a patient with mixed-phenotype acute leukemia who developed *C. perfringens* sepsis in the context of profound chemotherapy-induced neutropenia.

## Introduction

*Clostridium* species are anaerobic Gram-positive rod bacteria, ubiquitous in nature, and found on the normal flora of the human gastrointestinal tract. They can cause a broad spectrum of pathological conditions, ranging from food poisoning and bacteremia to gas-gangrene and necrotizing enterocolitis. *Clostridium perfringens* (*C. perfringens*) is the main representative of this group of pathogens and has been associated with severe and fatal sepsis, especially in patients with diabetes mellitus, recent instrumentation of the digestive tract, and cancer, typically acute leukemias and gastrointestinal carcinomas [1,2]. In fact, malignancy, immunosuppression, and neutropenia seem to be the most frequent underlying conditions, and gastrointestinal and hepatobiliary sites the most common sources of bacteremia. Despite these risk factors, anaerobic bacteremia is uncommon, and only around 2% of positive blood cultures are due to a *Clostridium* species [3].

The mechanisms through which *C. perfringens* causes fulminant disease are its short doubling time (approximately seven minutes) and ability to generate, among others, alpha-toxin, a phospholipase C lecithinase that directly hydrolyses the cell membrane [4]. This leads to severe necrosis, depending on the affected tissue, and massive intravascular hemolysis, associated with a 74% mortality rate [2,4]. This syndrome can be challenging to diagnose due to its rapid clinical course, frequently leading to death even before *C. perfringens *can be identified on blood cultures [1].

In patients with hematological malignancies, such an infection can be fatal in a matter of hours due to refractory shock [5]. Acute leukemias are typically associated with neutropenia and require highly immunosuppressive treatments, which further predisposes patients to bacterial infections. These patients require close vigilance, urgent clinical assessment, and empirical broad-spectrum antibacterial agents in case of febrile neutropenia, a possible harbinger of septic shock if left inadequately treated.

We present the case of a patient on treatment for mixed-phenotype acute leukemia who developed liver abscess, intravascular hemolysis, and septic shock caused by *C. perfringens *in the setting of profound neutropenia.

## Case presentation

A 62-year-old woman with a history of mixed-phenotype acute leukemia was admitted as an inpatient in the Hemato-Oncology Unit of the Centro Hospitalar Universitário de São João for first-line consolidation treatment with intensive chemotherapy with fludarabine, cytarabine, filgrastim, and idarubicin (FLAG-Ida) and sorafenib.

She underwent induction treatment one month before with the same regimen, achieving complete remission. During that hospital stay, she had two separate episodes of febrile neutropenia. The patient initially spiked a fever on admission and was treated with piperacillin/tazobactam and vancomycin; no infectious sources were identified and temperature soon abated after the start of the antimicrobials. However, the fever recurred after the antimicrobials were stopped, and piperacillin/tazobactam was restarted. The patient remained febrile and a piperacillin/tazobactam-resistant strain of *Klebsiella oxytoca *(*K. oxytoca*) was identified in blood cultures, motivating escalation to meropenem. The patient improved and meropenem was discontinued after seven days of treatment and after new blood cultures were negative for the previously identified pathogen. She was discharged home while clinically well and after recovery from neutropenia.

Twelve days after discharge, the patient was readmited for consolidation treatment to a ward with positive air pressure and air flow control in the Hemato-Oncology Unit. As per hospital protocol, antiviral and antifungal prophylaxis were instituted with acyclovir and posaconazole, respectively; no routine prophylaxis is given for bacterial infections or for *Pneumocystis jirovecii* (*P. jirovecii*) pneumonia in our center.

Eleven days after the start of chemotherapy, the patient developed febrile neutropenia while hemodynamically stable. Piperacillin/tazobactam was started empirically, but blood cultures again showed a *K. oxytoca* bacteremia with a resistant strain. She was immediately changed to meropenem and responded well, and was later de-escalated to cefepime to complete seven days of treatment.

After this episode had resolved, 25 days after the start of chemotherapy and while the patient was profoundly neutropenic (white blood cell count 0.06x10^9^/L), a new febrile syndrome developed. There were no other signs of a specific source for infection and she remained hemodynamically stable, and so blood (both peripheral and central venous catheter) and urine cultures were obtained while meropenem was initiated as empiric broad-spectrum antibiotic therapy for febrile neutropenia, considering previous findings of a recurrent *K. oxytoca* resistant to piperacillin/tazobactam. Analytically, and comparing to routine blood work from the day before, a sudden drop in hemoglobin concentration from 8.6 to 6.8 g/dL (reference range 12.0-16.0) and a new-onset unconjugated hyperbilirubinemia (5.54 mg/dL in total bilirubin, reference range <1.2 mg/dL) were noted. Remarkably, both potassium and lactate dehydrogenase (LDH) concentrations were unreportable due to hemolysis.

**Figure 1:**
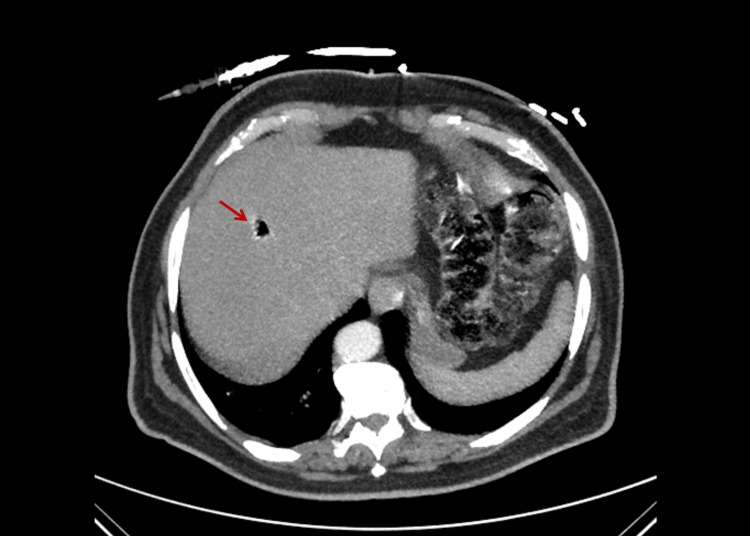
Gas-filled liver abscess on abdominal CT scan. Well circumscribed air-dense lesion with 13 mm in diameter in the right hepatic lobe (arrow), without contrast-enhanced areas in the surrounding parenchyma, association to vascular structures, or pneumobilia. Although initially interpreted as an artifact, the lesion is more suggestive of an abscess caused by a gas-producing pathogen since it was confirmed on additional imaging tests.

A few hours later that day, the patient was agitated and presented general malaise. She was somnolent, complained of unspecific abdominal discomfort, and showed a decrease in oxygen saturation. The urine presented frank red discoloration. A new analytic study showed rapidly aggravating anemia (Hb 4.5 g/dL) and new-onset microcytosis (MCV {mean corpuscular volume} 76.4 fL, reference range 87.0-103.0, previously 88.9 fL), associated with metabolic acidosis (pH 7.14, reference range 7.35-7.45), marked increase in serum lactate (19.5 mmol/L, reference range <2.0) and acute kidney injury (creatinine 1.43 mg/dL, reference range 0.51-0.95, previously 0.66). Unfortunately, a peripheral blood smear was not obtained. A contrast-enhanced computed tomography (CT) of the thorax and abdomen was performed to exclude signs of internal bleeding, considering the findings of worsening anemia, transfusion-dependent thrombocytopenia, and red-colored urine. The scan was otherwise unremarkable except for an air-dense lesion on the right hepatic lobe, not typical of pneumobilia and without any apparent association with vascular structures (Figure 1). No abnormalities were reported in the urinary tract.

The patient was kept on aggressive hydration and multiple transfusions with packed red blood cell concentrates. She was transferred to the intensive care unit (ICU) due to progressive clinical and neurological deterioration and for supportive treatment of a neutropenic septic shock with respiratory, renal, hepatic, and neurological dysfunction, and vancomycin was added to meropenem for additional large-spectrum antibacterial coverage with close monitoring of vancomycin levels according to protocol; considering hospital epidemiology, broader coverage for Gram-negative bacteria was felt unnecessary. A urine sample was obtained after admission to the ICU, and erythrocyturia (79.3 cells/µL, reference range <27.0) was abnormally mild for a hematuria-like red discoloration. This information, together with a rapidly progressing acute microcytic anemia, the apparent absence of internal or external bleeding, and the presence of an air-dense hepatic lesion associated with fever, was suggestive of massive intravascular hemolysis with probable hemoglobinuria and a gas-forming liver abscess, pointing to a toxin-producing clostridial infection. A few days after ICU admission, the initial blood cultures allowed the identification of an extended-spectrum beta-lactamase (ESBL)-negative multidrug-sensible *Escherichia coli* (*E. coli*) and concomitant co-infection with Gram-positive rods of *C. perfringens*, a known alpha-toxin producing pathogen, confirming the clinical suspicion.

During her stay in the ICU, the patient was started on vasopressors for the sepsis-related cardiovascular dysfunction, and on continuous veno-venous hemofiltration for failing to improve her kidney function. Antibacterial treatment was initially effective and a reduction in hemolytic markers was seen, as the patient also reduced her red cell transfusion requirements. Despite rapidly weaning the vasopressors and transitioning to sustained low-efficiency dialysis with support from the Nephrology team, respiratory insufficiency aggravated with increasing oxygen demands, which prompted the ICU team to start high-flow nasal cannula therapy and to repeat a CT scan of the thorax. Due to the presence of bilateral consolidations with ground-glass appearance, the patient was empirically started on amphotericin B for a possible invasive fungal infection. In order to obtain microbiological evidence for this, a bronco-alveolar lavage was performed after discussion with the Pneumology team. Both fungal cultures and markers were negative, including galactomannan on the blood and on the lavage; beta-D-glucan quantification is not available at our center. A polymerase chain reaction (PCR) assay for *P. jirovecii* was found to be positive and, although the radiological image was not highly suggestive of this diagnosis, the patient was started on co-trimoxazol and on a course of corticosteroids after a multidisciplinary meeting between the Hematology, ICU, and Infectious Diseases teams. Meropenem was maintained for twelve days and vancomycin was later deescalated to penicillin G directed at the hepatic abscess likely caused by the gas-producing anaerobe, aiming to more efficiently control the source of infection. Indeed, considering the complexity of this case, a multidisciplinary approach was necessary to adjust treatments on an almost daily basis.

The patient indeed survived the clostridial septic shock and the febrile neutropenia resolved. She was transferred back to the ward still on co-trimoxazole but improving from the pneumocystosis and no longer requiring oxygen support. Although the hepatic air-dense lesion disappeared on imaging re-evaluation, hyperammonemia, and other hepatic parameters failed to normalize and the patient did not recover from multifactorial kidney injury, permanently requiring dialysis. Two months after starting chemotherapy and more than a month after the septic shock, she further developed a progressive decline in awareness and a multifactorial metabolic encephalopathy ensued. The patient eventually died without hematopoietic recovery despite no evidence of relapsed leukemia.

## Discussion

This case stands as a rather unusual presentation of both *C. perfringens* sepsis and febrile neutropenia in a patient with mixed-phenotype acute leukemia undergoing FLAG-Ida, a highly myelosuppressive chemotherapy regimen. Although neutropenic patients frequently lack signs of infection other than fever, several signs recorded in this case point to this uncommon etiology. These are mainly due to the bacterial production of alpha-toxin, a phospholipase C lecithinase that hydrolyses the red cell membrane, producing intravascular hemolysis with microspherocytes and releasing hemoglobin to the circulation, which manifests as hemoglobinuria [4]. Indeed, the presence of rapidly aggravating microcytic anemia, hyperbilirubinemia, increase in LDH, hemoglobinuria, and the detection of hemolyzed blood samples during a new-onset febrile episode should raise the suspicion of this toxin-producing anaerobe [4]. The peripheral blood smear could also add valuable information for the differential diagnosis since anisocytosis, microspherocytes, and dehemoglobinized ghost cells are characteristic findings [6]. Unfortunately, the absence of a smear on the day of onset of the clostridial sepsis in this case probably delayed the diagnosis of hemolysis and could have helped steer the working diagnosis to a toxin-producing bacteria rather than a hemorrhagic shock with suspected hematuria. Whether this would have been enough to prevent the patient from undergoing a contrast-enhanced CT scan is doubtful, since bleeding is a far more frequent complication than *C. perfringens* sepsis in patients undergoing intensive chemotherapy for acute leukemia.

A retrograde biliary infection or transient bacteremia from an intestinal source could explain the formation of a gaseous liver abscess by an anaerobe found in the intestinal flora [2,7], and considering the frequent mucosal damage seen in neutropenic patients undergoing chemotherapy, increased gut permeability is likely a contributing factor. Interestingly, polymicrobial infections are seen in 60% of cases of clostridial bacteremia in cancer patients, with most of the accompanying bacteria being commonly found on the gastrointestinal tract [3], like *E. coli* in this case. Since no food poisoning cases were recorded in our hospital and there was no history of penetrating trauma or gastrointestinal malignancy, which are known risk factors for this type of infection, severe neutropenia is likely to account for the spontaneous clostridial sepsis in this case. The relative contribution of *E. coli* to the outcome seen in this patient should not be overlooked, and bacteremia by this agent is a frequent cause of febrile neutropenia and septic shock in patients with hematological malignancies. However, some of the most dominant features presented here, including hemolysis and liver dysfunction, were most likely caused by *C. perfringens*.

Unlike most reports of massive intravascular hemolysis due to *C. perfringens*, where the median time from patient admission and death is 9.7 hours [4], in this case, the patient survived the acute phase of this fulminant infection. Since she was already an inpatient, the rapid detection of fever, initiation of broad-spectrum antibacterial therapy, aggressive support with blood products, and admission to the ICU were fundamental to prevent further deterioration of her clinical status [8]. Meropenem was chosen as first-line treatment for febrile neutropenia due to previous infections, and *C. perfringens* is typically susceptible to carbapenems and penicillins [9]. Although clindamycin has a role in preventing further production of clostridial toxins, treatment with this agent was not started due to the rapid resolution of hemolysis after admission to the ICU [4,9]. An additional therapeutic option in cases of gas gangrene is source control, which includes surgical debridement or drainage and is significantly associated with improved survival [3,4]. Due to the relatively small size of the liver abscess and its centralized location on the right lobe, as shown on the CT scan, and since the patient remained neutropenic and thrombocytopenic, this surgical approach was not considered [9].

Despite surviving the acute infection, multi-organ failure after the septic shock was not reversible. Renal dysfunction was likely associated with acute tubular necrosis due to shock and hemoglobinuria [10], but additional factors could have contributed to this outcome, including nephrotoxicity from the contrast used in the CT scan and vancomycin. Hepatic dysfunction with hyperammonemia also persisted, despite the apparent resolution of the hepatic abscess. Subsequent complications, like the bilateral pneumonia suggestive of pneumocystosis, further aggravated her clinical condition. The lack of hematopoietic recovery from chemotherapy-induced marrow aplasia, possibly a manifestation of hematological dysfunction secondary to several disease processes and co-trimoxazol treatment, helped to define a significantly complex and unfavorable scenario. Indeed, it is critical to provide immediate supportive care to prevent the devastating outcomes seen in acute *C. perfringens* sepsis on neutropenic patients, as initially managed in this case. However, even in that ideal scenario, hemolysis alone seems to increase the mortality rate of this infection to around 80% [1], and severe comorbidities arising afterward as a consequence of this highly aggressive bacterial sepsis can also permanently define its overall dismal prognosis.

## Conclusions

This clinical case highlights some of the most remarkable features of a very uncommon infection. Most of the classical signs and symptoms described for *C. perfringens* sepsis were seen in this highly immunosuppressed patient undergoing chemotherapy for acute leukemia, including massive intravascular hemolysis and a gas-forming abscess. The short bacterial doubling time and the highly aggressive nature of alpha-toxin in producing cell lysis explain the acute severe deterioration that patients experience just a few hours after the onset of fever. As a direct consequence of this pathophysiology, rapid identification of these clinical features is paramount. This is especially true for hemolytic anemia, which is not a feature commonly seen in the diagnostic approach of febrile neutropenia and can be overlooked or mistakenly interpreted as blood loss in patients with severe thrombocytopenia and sepsis; in this case, a simple blood smear could be of high value for the differential diagnosis. Improved outcomes may be achieved through effective antimicrobial therapy and organ support, although the aggressive nature of this syndrome seems to be, in most cases, clinically overwhelming.
